# Prognostic model for predicting overall survival in children and adolescents with rhabdomyosarcoma

**DOI:** 10.1186/1471-2407-14-654

**Published:** 2014-09-05

**Authors:** Limin Yang, Tetsuya Takimoto, Junichiro Fujimoto

**Affiliations:** Epidemiology and Clinical Research Center for Children’s Cancer, National Center for Child Health and Development, 2-10-1 Okura, Setagaya-ku, Tokyo, 157-8535 Japan; Division of Allergy, Department of Medical Subspecialties, Medical Support Center for Japan Environment and Children’s Study (JECS), National Center for Child Health and Development, 2-10-1 Okura, Setagaya-ku, Tokyo, 157-8535 Japan

**Keywords:** Rhabdomyosarcoma, Cancer, Nomogram, Overall survival

## Abstract

**Background:**

The purpose of this study was to develop a prognostic model for the survival of pediatric patients with rhabdomyosarcoma (RMS) using parameters that are measured during routine clinical management.

**Methods:**

Demographic and clinical variables were evaluated in 1679 pediatric patients with RMS registered in the Surveillance, Epidemiology, and End Results (SEER) program from 1990 to 2010. A multivariate Cox proportional hazards model was developed to predict median, 5-year and 10-year overall survival (OS). The Akaike information criterion technique was used for model selection. A nomogram was constructed using the reduced model after model selection, and was internally validated.

**Results:**

Of the total 1679 patients, 543 died. The 5-year OS rate was 64.5% (95% confidence interval (CI), 62.1-67.1%) and the 10-year OS was 61.8% (95%CI, 59.2-64.5%) for the entire cohort. Multivariate analysis identified age at diagnosis, tumor size, histological type, tumor stage, surgery and radiotherapy as significantly associated with survival (p < 0.05). The bootstrap-corrected c-index for the model was 0.74. The calibration curve suggested that the model was well calibrated for all predictions.

**Conclusions:**

This study provided an objective analysis of all currently available data for pediatric RMS from the SEER cancer registry. A nomogram based on parameters that are measured on a routine basis was developed. The nomogram can be used to predict 5- and 10-year OS with reasonable accuracy. This information will be useful for estimating prognosis and in guiding treatment selection.

## Background

Rhabdomyosarcoma (RMS) is the most common soft-tissue sarcoma in children and adolescents and accounts for 3% of all pediatric tumors [1]. Approximately 350 children are diagnosed with RMS in the United States every year [2]. Incidence peaks at a very young age. Because RMS is derived from immature striated skeletal muscle, this disease can occur at any site in the body. Prognosis of RMS has improved significantly, with multidisciplinary management accounting for most of the increase in survival rate. Since 1972, the Intergroup Rhabdomyosarcoma Study Group (IRSG) has conducted a series of clinical trials and published a series of treatment guidelines for different primary sites. As a result, the long-term survival rate of these patients has nearly tripled from approximately 25% in 1970 to more than 70% in the 1990s [3, 4].

The rarity of this disease means that most information regarding survival is derived from these clinical trials. However, overall survival (OS) results differ between clinical trials and population-based cancer registries because of important differences between patients treated in routine practice and those treated in clinical trials. For example, IRSG reports showed a 5-year OS of 70% in the 1990s [3, 4], while, even in the 2000s, the 5-year OS was only approximately 50% in children with RMS according to population-based data [5]. Clinical trials may select participants based on strict inclusion criteria, which consider the extent of disease, previous history of treatment, comorbidities, psychosocial conditions and other factors [6]; patients in a poor condition may thus be excluded from the protocol. OS in trials may therefore not reflect the prognosis of patients who receive treatment in a community setting.

Individualized estimation of the prognosis could be useful for counseling cancer patients on treatment selection and for optimizing therapeutic approaches [7]. However, to the best of our knowledge, there is currently no such estimation tool for RMS based on patients from the general population. In this study, we analyzed the OS in children and adolescents with RMS using population-based data collected by the Surveillance, Epidemiology, and End Results (SEER) program of the National Cancer Institute (NCI) [5], and constructed a nomogram based on variables collected from the routine cancer registry, with the aim of providing clinicians and patients with a practical clinical tool to predict survival.

## Methods

### Study population

The data were derived from the SEER program, which collects demographic, diagnostic and treatment information on all newly diagnosed cancer patients residing within specific US geographic regions. Registry data are submitted without personal identifiers to the NCI, and these data are publicly available for research purpose. Because all information in public-use SEER database remains de-identified, approval by an ethics committee was not necessary to perform the analysis [8]. All authors have signed the data-use agreement and got permission from SEER program to use this data.

**Figure 1:**
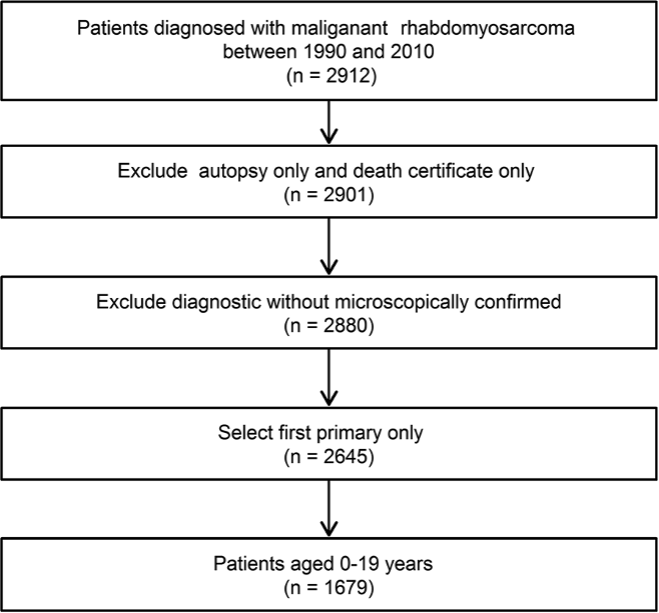
**Flow chart for the creation of the Surveillance, Epidemiology, and End Results (SEER) data set.**

Using the SEER registry public database, we identified patients with RMS diagnosed from 1990 to 2010 [5]. Children diagnosed with malignant, first primary RMS and aged 0–19 years were eligible for this analysis. In this study, eligible RMS cases had International Classification of Childhood Cancer (ICCC) code IXa, corresponding to ICO-O-3 morphology codes: 1) RMS not otherwise specified 8900/3; 2) pleomorphic RMS adult-type 8901/3; 3) mixed-type RMS 8902/3; 4) embryonal RMS 8910/3; 5) spindle cell RMS 8912/3; 6) alveolar RMS 8920/3; or 7) embryonal sarcoma 8991/3. Patients were excluded from the analysis if the diagnosis was made at autopsy or by death certificate only. Patients with no confirmation of diagnosis by microscopy were also excluded. After selection, there were 1679 cases left in the cohort. The flow chart for data selection is shown in Figure 1.

### Data analysis

In the description of variables and calculation of OS, age at diagnosis was classified as 0–4, 5–9, 10–14 or 15–19 years. Age at diagnosis was treated as a continuous variable in multivariate analysis. Other clinical factors included primary tumor site, histologic tumor subtype, tumor stage, tumor size, surgery and radiotherapy (RT). Primary tumor sites were classified as favorable or unfavorable based on the criteria for staging of pediatric tumors [9]. The head and neck (nonparameningeal), genitourinary (non-bladder/prostate), and bile duct regions were defined as favorable sites, all other sites were defined as unfavorable, and an unknown site was regarded as a missing value. Histology was classified as embryonal, alveolar or other histological subtype. Histological subtypes with RMS not otherwise specified were treated as missing values. Tumor stage was classified according to the SEER historic staging system. Cases with insufficient information to define the stage were regarded as having a missing value. Tumor size was truncated at 20 cm and was grouped into three levels for both character description and calculation of OS: 1) 0–4 cm; 2) 5–9 cm; and 3) ≥10 cm. Size was treated as a continuous variable in the multivariate model.

### Statistical methods

All missing values were imputed with the ‘transcan’ function of the rms package [10]. OS was calculated by the Kaplan-Meier product-limited method. Survival curves were compared using the log-rank test. Cox proportional hazard regressions were performed to assess the effects of covariates on OS. For continuous variables, we fitted restricted cubic splines with three knots at 10%, 50% and 90% empirical quantiles. We also considered the interaction effect between surgery and RT. The proportional hazard assumption was justified by examining residual plots. The Akaike information criterion was utilized for model selection. We constructed a nomogram with the beta coefficients of variables in the reduced model.

The model was internally validated. We generated 200 bootstrap samples to determine the calibration and discrimination of the model. Calibration refers to the ability of a model to make unbiased estimates of outcome. Calibration was assessed using a calibration curve generated by plotting the model-predicted 5-year and 10-year survival probabilities against the observed probability, as calculated by the Kaplan-Meier method. The prognostic accuracy of the model was quantified by computing the concordance index (c-index) described by Harrell et al. [11]. The c-index is a discrimination measure that estimates the probability that, of two randomly chosen patients, the patient with the higher predicted survival will outlive the patient with the lower predicted survival. The c-index ranges from 0.5 (no discrimination) to 1.0 (perfect discrimination).

All statistical analyses were conducted using R version 3.1.0 software (Institute for Statistics and Mathematics, Vienna, Austria; http://www.r-project.org) [12]. The model and nomogram were constructed using the R package rms [10]. All statistical tests were two-sided, and values of p < 0.05 were considered significant.

## Results

Patient demographics are listed in Table 1. A total of 1679 pediatric patients with RMS were included in the study. Approximately 38.1% of the subjects were aged 0–4 years, 23.2% were 5–9 years, 20.6% were 10–14 years and 18.1% were 15–19 years. There were 974 (58.0%) boys, and 705 (42.0%) girls. The majority of patients were white (75.9%). Approximately 61.1% of RMS occurred at unfavorable sites. Around 59.0% of patients were diagnosed with embryonal RMS, 33.2% with alveolar RMS and 7.7% with other RMS. Based on SEER staging, 33.4% of patients had localized tumors, 34.9% had regional RMS and 31.7% had metastasis. More than half (62.8%) of the patients had received RT, and 59.1% received surgery.

**Table 1:** **Patient demographics and overall survival**

	All patients		5 Years OS (%)		10 Years OS (%)		p
Characteristics	No.	Events	Rate	95%CI	Rate	95%CI	
Entire cohort	1679	543	64.5	62.1-67.1	61.8	59.2-64.5	
Age (years)							<0.001
0-4	639	173	71.3	67.5-75.2	69.1	65.2-73.2	
5-9	390	97	73.2	68.5-78.2	68.8	63.6-74.5	
10-14	346	134	56.4	50.9-62.6	52.4	46.6-59.0	
15-19	304	139	47.9	42.1-54.6	47.3	41.5-54.0	
Tumor size (cm)							<0.001
0-4	618	116	79.5	76.0-83.1	77.1	73.4-81.1	
5-9	675	237	61.6	57.7-65.7	57.6	53.5-62.1	
≥10	386	190	45.9	40.7-51.7	44.4	39.1-50.3	
Sex							0.311
Male	974	306	65.7	62.5-69.1	62.5	59.1-66.1	
Female	705	237	63.0	59.2-67.0	60.8	56.8-65.0	
Race							0.359
White	1274	407	65.2	62.4-68.1	62.2	59.2-65.3	
Black	277	88	64.0	58.0-70.7	62.2	55.9-69.2	
Others	128	48	58.7	50.0-69.0	56.6	47.5-67.5	
Site							<0.001
Unfavorable	1026	406	56.6	53.2-59.9	53.5	50.1-57.1	
Favorable	653	137	77.1	73.6-80.7	74.6	70.8-78.5	
Stage							<0.001
Localized	561	83	84.0	80.7-87.5	81.1	77.3-85.1	
Regional	586	152	72.4	68.6-76.5	68.5	64.3-73.1	
Distant	532	308	35.7	31.5-40.5	34.4	30.1-39.2	
Histology							<0.001
Embryonal	991	249	73.5	70.6-76.5	70.8	67.6-74.1	
Alveolar	558	263	46.3	41.9-51.2	43.2	38.7-48.3	
Others	130	31	73.1	64.9-82.3	71.4	62.9-81.0	
Surgery							<0.001
None	686	294	52.0	48.0-56.2	50.4	46.4-54.7	
Surgery	993	249	73.2	70.2-76.3	69.9	66.3-73.0	
Radiotherapy							0.045
None	625	213	62.7	58.7-67.0	60.8	56.6-65.3	
Radiation	1054	330	65.6	62.5-68.9	62.3	59.1-65.8	

OS, overall survival; CI, confidence interval.

The 5-year OS rate for the entire cohort was 64.5% (95% confidence interval (CI), 62.1-67.1%) and the 10-year OS rate was 61.8% (95%CI, 59.2-64.5%). Five- and 10-year OS rates by characteristic are listed in Table 1. Sex and race had no influence on OS. Prognosis worsened with increasing age; young children (0–4 years) had a better prognosis than adolescents (15–19 years), with 5-year OS of 71.3% and 47.9%, respectively. Children with embryonal RMS had a longer survival than those with alveolar RMS, with estimated 5-year OS of 73.5% and 46.3%, respectively. Patients with localized tumors had a better prognosis (5-year OS of 84.0%) than those with regional disease (72.4%) or distant metastasis (35.7%). RMS at favorable sites had a better prognosis than that at unfavorable sites (p < 0.001). Patients with surgery had improved survival compared with those without surgery (p < 0.001). RT showed a weak but significant association with prognosis; 5-year OS was 65.6% in patients with RT compared with 62.7% in those without RT (p = 0.045).

Multivariate analysis was performed using a Cox proportional hazards regression model. We pre-specified nonlinearity for age at diagnosis and tumor size variables, and considered the effect on prognosis of the interaction between surgery and RT. Residual plots indicated that the proportional hazards assumption held. After model selection, we obtained a reduced model. Beta coefficients and hazard ratios of variables are listed in Table 2.

**Figure 2:**
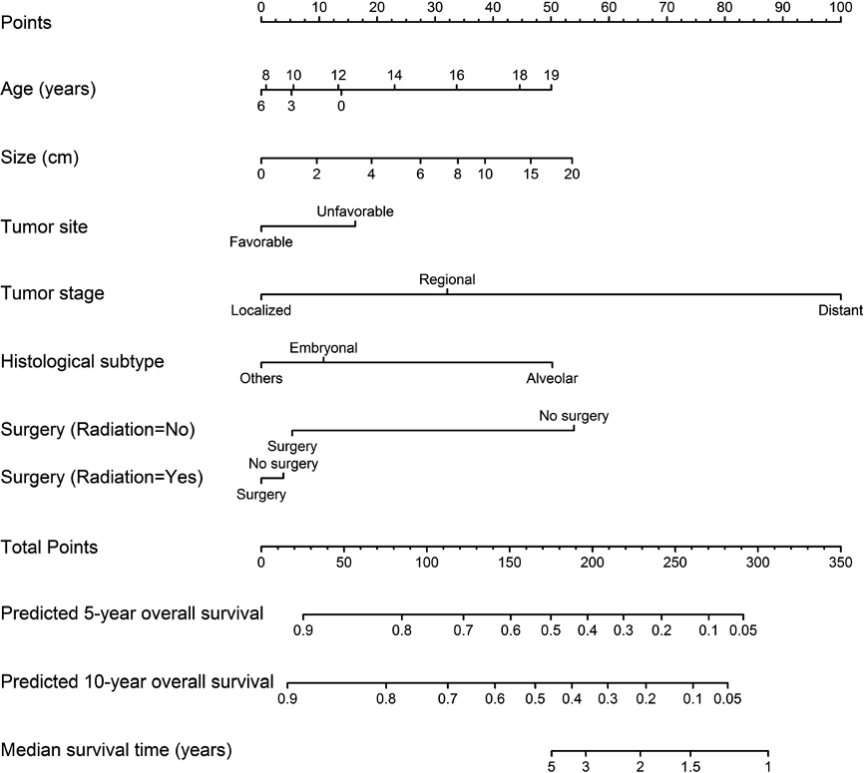
**Nomogram for predicting 5- and 10-year overall survival and median survival time.** Instructions: Locate the patient’s characteristic on the variable row, draw a vertical line straight upward to the points row to obtain a points value for the variable. Move to the next row of variables, and repeat this process to get points for each variable. Sum the total points and drop a vertical line from the total points row to assign the values for overall survival rates.

The nomogram included age at diagnosis, size, tumor site, stage, histological type, surgery and RT (Figure 2). To use the nomogram, we drew a vertical line to the point row to assign point values for each variable, summed the point values for each variable to obtain total points, and then dropped a vertical line from the total points row to get the 5- and 10-year OS rates.

**Figure 3:**
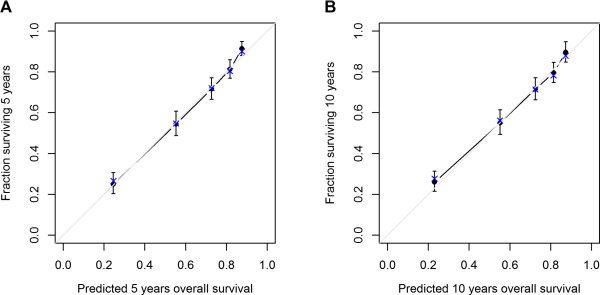
**Calibration plot. (A)** Five-year overall survival; **(B)** 10-year overall survival. The grey line is the “ideal” line if there is a perfect match between predicted and observed survivals. Vertical arrows represent 95% confidence intervals of observed survival. Dots correspond to apparent predictive accuracy. X marks the bootstrap-corrected estimates.

The model was internally validated. Discrimination suggested good accuracy with a bootstrap-corrected c-index of 0.74, which denotes 74% probability that, of two randomly selected patients, the patient who survives longer will have a higher survival probability than the patient with shorter survival. The calibration plots for 5- and 10-year OS are shown in Figure 3. Points in the calibration plot were close to the 45° line, which suggested that the model was well-calibrated for all predictions.

**Table 2:** **Cox proportional hazards multivariate regression model parameters**

Covariate	Beta coefficient	Hazard ratio	95% CI	p
Age	-0.037*	-**	-	0.154
Age’	0.089*	-**	-	0.013
Size	0.006^†^	-^††^	-	0.095
Size’	-0.004^†^	-^††^	-	0.359
Favorable site	-0.204	0.82	0.65-1.02	0.076
Stage				
Regional	0.404	1.50	1.13-1.98	0.004
Distant	1.259	3.52	2.64-4.70	<0.001
Histology				
Alveolar	0.497	1.64	1.35-2.00	<0.001
Other	-0.135	0.87	0.59-1.29	0.499
Received surgery	-0.612	0.54	0.40-0.72	<0.001
Received RT	-0.632	0.53	0.42-0.68	<0.001
Interaction terms				
Surgery × RT	0.564	1.75	1.24-2.50	0.002

CI, confidence interval; RT, radiotherapy.

*Age was modeled using a restricted cubic spline function with three knots, which yields two independent beta coefficients, annotated as Age and Age’.

**The hazard ratio varies continuously with age.

^†^Size was modeled using a restricted cubic spline function with three knots, which yields two independent beta coefficients, annotated as Size and Size’.

^††^The hazard ratio varies continuously with size.

## Discussion

The current study evaluated OS among pediatric patients with newly-diagnosed RMS in a population-based dataset, and constructed a nomogram to predict 5- and 10-year OS. This prognostic tool will be useful for estimating prognosis and guiding treatment selection.

The rarity of this disease means that most published studies are retrospective analyses of clinical studies, or small, single-institution, observational studies. Results from a single institution often fail to identify a true relationship between outcome and risk factors because of the small sample size and short follow-up period. Our analyses were based on the SEER database, which is considered to be the largest cancer registry. Reports from a population-based cohort have the advantage of including many more patients, thus increasing the power to estimate the true effects of risk factors on survival. Moreover, unlike most results from clinical studies, analysis of a population-based database includes not only those treated using formal protocols, but also those excluded from protocols because of comorbidity, tumor stage, or other factors. The reported results thus represent the full spectrum of the disease. Furthermore, SEER data are high quality and are collected in a uniform manner with uniform data standards. Quality control ensures that the SEER program has a relatively low rate of errors in the cancer registry [6].

Our study cohort included 1679 RMS patients and 543 deaths, which sample size was adequate to establish a reasonable model. The outcome measure of OS is one of the most useful pieces of information for counseling and is commonly used to develop staging schemes. Although the nomogram is not perfectly accurate, the error bars in the calibration plot suggest that predictions from the nomogram are within approximately 5% of the actual probability on average, and the bootstrap-corrected c-index of 0.74 suggests that the nomogram has good ability to discriminate among patients. This accuracy is comparable with most published nomograms for cancer prognosis.

The prognostic nomogram is a model-based tool to predict patient outcome. It directly quantifies the prognosis of individual patients based on proven prognostic factors. Different from a staging or scoring system, a nomogram considers multiple commonly available prognostic variables simultaneously, including continuous variables. Individual predictions are expressed on a probability scale, making it more easily understood by patients and clinicians than relative rates or hazard ratios [13]. A nomogram has the potential to stratify patients for clinical studies, meaning that treatment regimens can be tested in more homogenous populations. Selecting high-risk patients based on predictions from a nomogram can also help to improve trial efficiency; for example, trials evaluating a treatment strategy could target patients with poor prognoses. Identifying high-risk patients for trial recruitment using a nomogram will increase the power to detect differences among treatment effects, thus reducing the required sample size. This method has been used in prospective randomized trials [14].

There is increasing interest in personalized medicine. A number of cancer nomograms to predict prognosis have been published in recent decades, such as for prostate, breast, soft-tissue sarcoma, and other cancers, including our previous nomogram for thyroid cancer [7, 15–23]. To the best of our knowledge however, the nomogram constructed in this study represents the first OS nomogram for pediatric RMS that is generalizable to the population.

The results of the Cox model identified age at diagnosis, stage of tumor, tumor size, histological subtype and treatment as important predictors of RMS survival in pediatric patients. The findings are expressed consistently in the nomogram. For example, adolescence, distant disease, large tumor size, alveolar RMS and no treatment, which were associated with a reduced survival based on the model, were given larger points in the nomogram. Meanwhile, a larger total point indicates a lower OS.

Simplicity is a strength of our model. Unlike models that aim to identify associations between prognosis and risk factors, predictive models should focus more on accuracy and parsimony [24]. Complex models including a number of variables may be abandoned in clinical practice. In contrast, the nomogram developed in this study relies on limited variables that are routinely available from the tumor registry, making it easy for clinicians to use to calculate survival for individual patients.

Adult RMS was not included in this model. Pediatric and adult RMS have different clinical characteristics and prognoses. For example, pleomorphic RMS is common among adult patients, but is seldom seen in pediatric patients. Additionally, adult patients have a poorer response to chemotherapy. Research has suggested that increased levels of a resistance-related protein in adult embryonal and pleomorphic RMS compared with pediatric RMS may explain the reduced response to chemotherapy [25]. Information regarding chemotherapy and variables in the protein level was not available in the current study and it was therefore not possible to adjust for these potential effects on prognosis in the SEER cohort. Moreover, adult RMS may have lower pathologic accuracy compared with pediatric RMS [9]. We therefore excluded adult RMS from the current analysis to avoid these confounders and bias and to increase the accuracy of the model.

Although our nomogram showed reasonable accuracy for predicting OS, care should be taken when using a nomogram for counseling. Because it is impossible to include all risk factors, the prognostic predictive value of a nomogram should not be used as the sole basis for selecting a treatment regimen; treatment should be selected based on not only the expected value from the nomogram, but also taking into account other prognostic factors and quality of life.

There were some limitations to our study. First, the SEER public dataset does not include information on chemotherapy, comorbidity and surgical margins, which are viewed as important prognostic variables. This information would be useful for refining the predictive model. Second, although we restricted our cohort to patients diagnosed after 1990, the study period still spanned approximately two decades, during which time there have been improvements in surgery, chemotherapy and RT. Our nomogram thus tends to underestimate current OS. Third, unlike IRSG clinical trials, the SEER program does not utilize a central pathology review to minimize misclassification [6]. Finally, we used internal validation to evaluate the accuracy of the model, and external validation based on independent data would be useful to validate the model further.

## Conclusions

In conclusion, we used a population-based dataset to establish and internally validate a model to estimate the probability that a pediatric patient will be alive 5 and 10 years after being diagnosed with RMS. This study represents an objective analysis of all currently available data from the SEER cancer registry. The model shows good ability to discriminate among patients, with a c-index of 0.74. This predictive tool may be useful for patient counseling and to enable more individualized treatment planning.

## Abbreviations

RMS: Rhabdomyosarcoma
SEER: Surveillance, Epidemiology, and End Results
OS: Overall survival
NCI: National Cancer Institute
IRSG: Intergroup Rhabdomyosarcoma Study Group
ICCC: International Classification of Childhood Cancer
c-index: Concordance index.
